# Two-dimensional Periapical, Panoramic Radiography Versus Three-dimensional Cone-beam Computed Tomography in the Detection of Periapical Lesion After Endodontic Treatment: A Systematic Review

**DOI:** 10.7759/cureus.7736

**Published:** 2020-04-19

**Authors:** Delphine P Antony, Toby Thomas, MS Nivedhitha

**Affiliations:** 1 Conservative Dentistry and Endodontics, Saveetha Dental College-Saveetha University, Chennai, IND

**Keywords:** periapical, panoramic radiograph, cbct, periapical lesion, endodontic treatment

## Abstract

Radiographic imaging is a common resource for endodontic diagnosis, treatment, and prognosis. Two-dimensional (2D) periapical and digital panoramic radiographs often showed image distortion; this issue was resolved with the emergence of three-dimensional (3D) cone-beam computed tomography (CBCT). This review examines the accuracy of various radiographic techniques in the assessment of periapical lesion after endodontic treatment. Our goal was to determine whether a 2D radiograph (periapical and panoramic) is as accurate as a 3D radiograph (i.e., CBCT) in the assessment of periapical lesion after endodontic treatment. We searched the electronic databases Medline and Cochrane and trial registries for ongoing trials. We included both retrospective and prospective studies comparing the efficacy of periapical healing with various radiographic techniques after endodontic treatment. The outcome of interest was the percentage detection of periapical lesions and periapical healing assessment after endodontic treatment. All data were collected using a specially designed extraction form. We assessed the risk of bias in the studies using the Cochrane tool for diagnostic tests (QUADAS). We judged two studies to be at low risk and two to be at moderate risk of bias. Although there was a difference in the percentage detection of periapical healing efficacy by various radiographic techniques, all studies reported that CBCT had higher accuracy in the detection of periapical lesions compared to periapical and panoramic radiography. The next best choice is periapical radiographs, followed by panoramic radiographs as they provide better visualization and accuracy.

## Introduction and background

Apical periodontitis (AP) is an inflammation of the periodontium caused by trauma, irritation, or infection through the root canal, regardless of whether the pulp is vital or non-vital. It represents the main indication for root canal treatment. Most patients with AP are asymptomatic. However, pain, tenderness to biting pressure, percussion, or palpation, as well as swelling are typical clinical expressions of symptomatic AP. The assessment of periapical status through radiographic examination is important because it may help to define treatment needs and relates treatment outcomes to various technical and clinical factors of the endodontic intervention. The radiographic assessment of AP is done using the periapical index (PAI). The PAI represents an ordinal scale of five scores ranging from no disease to severe periodontitis with exacerbating features [1]. The main objectives of endodontic treatment are to retain the normal function of the treated tooth and to prevent or heal AP [2,3]. A radiological examination is a major tool in dentistry for a thorough exploration that helps to achieve the goals mentioned above.

There are several types of radiographic interventions. Periapical radiography offers important evidence on the progression, regression, and persistence of AP [4]. This can confirm the number of roots and their configuration together with the presence or absence of periapical lesions and their locations. It corresponds to a two-dimensional (2D) aspect of a three-dimensional (3D) structure [5]. Panoramic radiography is a curved plane tomographic radiographic technique that obtains an image by synchronous rotation of the X-ray source and image receptor around the stationary patient. It helps in identifying anatomical landmarks, pathosis in maxilla, mandible and maxillary sinus [6]. Cone-beam computed tomography (CBCT) has been specifically designed to produce 3D images of the maxillofacial skeleton. CBCT is indicated for the diagnosis of pathosis of endodontic and non-endodontic origins, assessment of root canal morphology, evaluation of root and alveolar fractures, analysis of external and internal root resorption, and pre-surgical planning in root-end surgeries [7].

Periapical periodontitis in periapical radiography is represented as a reduction in mineral density that is represented as radiolucency. Lesions confined within the cancellous bone cannot be detected, whereas lesions with buccal and lingual cortical involvement produce distinct radiographic areas of rarefaction. To be visible radiographically, a periapical radiolucency should reach nearly 30-50% of bone mineral loss [8]. Panoramic imaging is similar to periapical radiographs but more useful for diagnostic problems requiring broad coverage of the jaws like large lesions [9]. The specific endodontic applications of CBCT involve the change from analog to digital imaging and advances in imaging theory and volume-acquisition data, enabling detailed 3D imaging. Investigations have demonstrated that a cyst could be distinguished from periapical granulomas by CBCT because it shows a marked difference in density between the content of the cyst cavity and granulomatous tissue, thus favoring a noninvasive diagnosis [10,11].

This review was conducted because radiographic imaging plays a vital role in the diagnosis, treatment plan, and prognosis of endodontic treatment of teeth with periapical periodontitis. If the 2D radiograph is shown to be as accurate as CBCT in the detection of periapical healing after endodontic treatment, it could be cost-effective and cost-beneficial for both the patient and dentist. The objective of the review is to determine whether periapical and panoramic radiograph is as accurate as CBCT in the detection of periapical lesion healing after endodontic treatment.

## Review

We included retrospective and prospective studies that compared periapical radiograph, panoramic radiograph, and CBCT. We excluded case reports, case series, animal studies, and in-vitro studies that measured the same outcomes of interest. Participants were aged 16 years or older who required root canal treatment for AP or apical lesions. All participants had at least one tooth (single-rooted or multi-rooted) with a history of secondary and primary endodontic infections as confirmed by a clinical examination.

The index tests were performed with conventional periapical and panoramic radiographs. Conventional radiographs used in all studies were obtained by paralleling technique and a horizontal angle difference of about 10 degrees. The films were processed in an automatic processor and developed by using standardized methods. The target conditions were AP and periapical lesions. The reference standard was 3D Accuitomo XYZ Slice View CBCT (J. Morita Corp., Osaka, Japan). The PAI scoring system was used in most of the included studies for radiographic assessment of AP by conventional radiography and CBCT.

For our search methods, we developed detailed strategies for each database to identify studies for this review. These were based on the search strategy developed for Medline Ovid and used a combination of controlled vocabulary and free text terms (a table with details of the search methods is provided in the Appendices). Our electronic searches were conducted on Medline and Cochrane. We did not place any restrictions on the language or date of publication when searching the electronic databases. For other resources, we searched trial registries for ongoing studies using the World Health Organization International Clinical Trials Registry Platform. Additionally, we manually searched, with the assistance of a librarian, the following journals: Journal of Endodontics; International Endodontic Journal; Oral Surgery, Oral Medicine, Oral Pathology, and Oral Radiology; and Endodontology.

**Figure 1:**
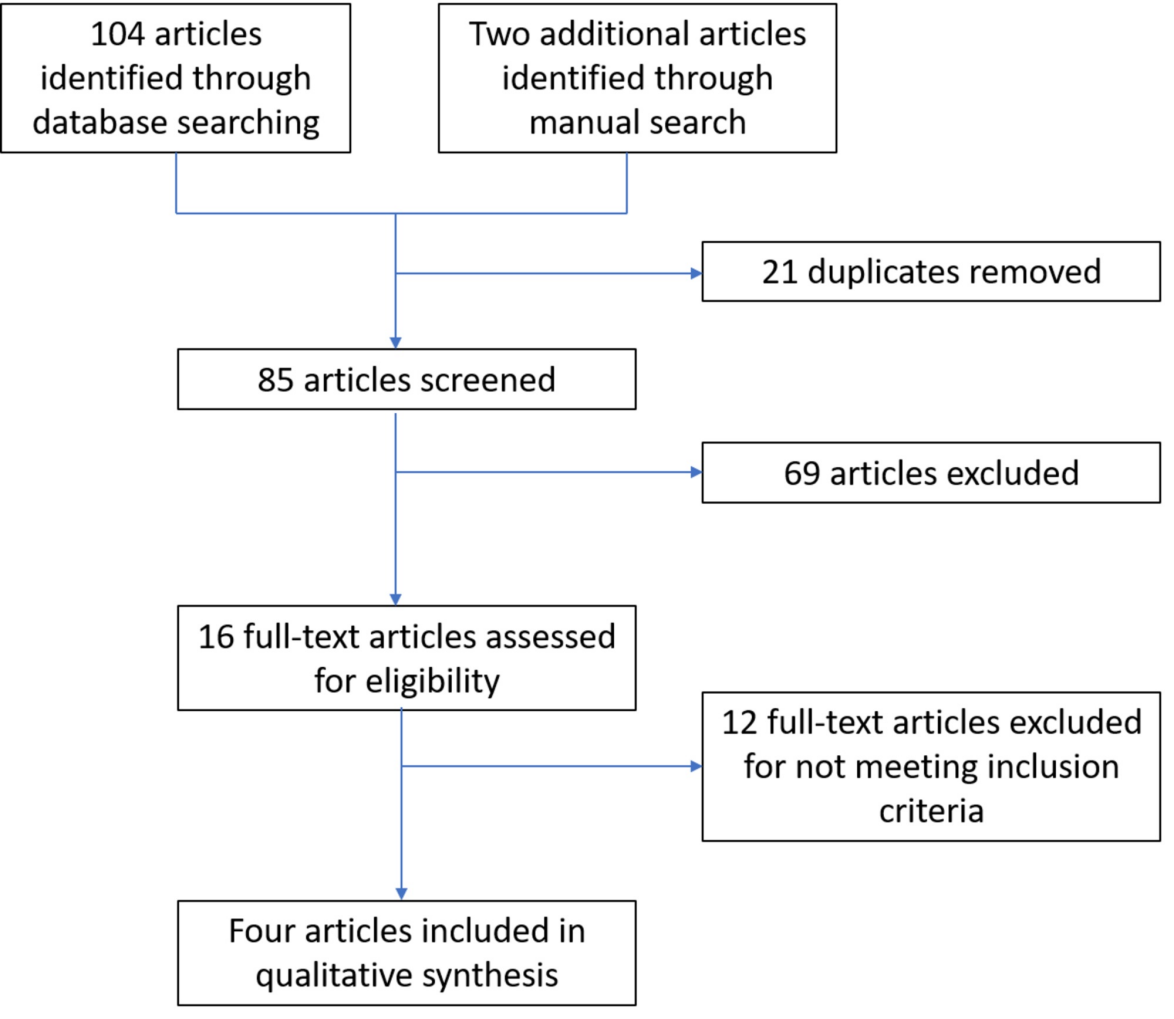
Study flowchart

The studies obtained by the search were assessed by the authors to determine whether studies met the selection criteria. All retrospective and prospective human in-vivo studies that compared periapical radiograph, panoramic radiograph, and CBCT for periapical periodontitis after endodontic treatment were included. Case reports, case series, animal studies, and in-vitro studies that measured the same outcomes of interest were excluded. We resolved any disagreements by discussion. The review authors independently examined the title and abstract of each article identified by the search strategy. Whenever studies appeared to meet the inclusion criteria for this review or where there was insufficient data in the title and abstract to make a clear decision, we obtained the full report. We also recorded the number of studies rejected at this or subsequent stages. A flowchart that summarizes the results of the search is given below (Figure 1).

Review authors independently extracted data using a specially designed data extraction form and entered them into a spreadsheet. For each study, we recorded the following data: year of publication; country of origin; study design; the number of participants included; the number of teeth evaluated; demographic details of the participants; study set-up; techniques used for index test and reference test; details of the outcomes reported, including method of assessment; and time(s) assessed. 

We performed an assessment of methodological quality. The review authors independently assessed the risk of bias of the included studies, and any disagreement was resolved through discussion and consensus. We used the recommended approach for assessing the risk of bias using QUADAS: a 14-item tool for the quality assessment of studies of diagnostic accuracy included in systematic reviews [12]. We addressed four domains for risk of bias and three domains for applicability of concerns, including patient selection, index test, reference standard, flow, and timing. Within each entry, we described what was reported to have happened in the study and monitored for insufficient detail to support a judgment about the risk of bias. We then assigned a judgment relating to the risk of bias for that entry of either low, high, or unclear. After considering the additional information provided by the authors, we summarized the risk of bias in the studies as one of the following: low risk of bias - a low risk of bias for all key domains; unclear risk of bias - an unclear risk of bias for one or more key domains; high risk of bias - a high risk of bias for one or more key domains.

**Figure 2:**
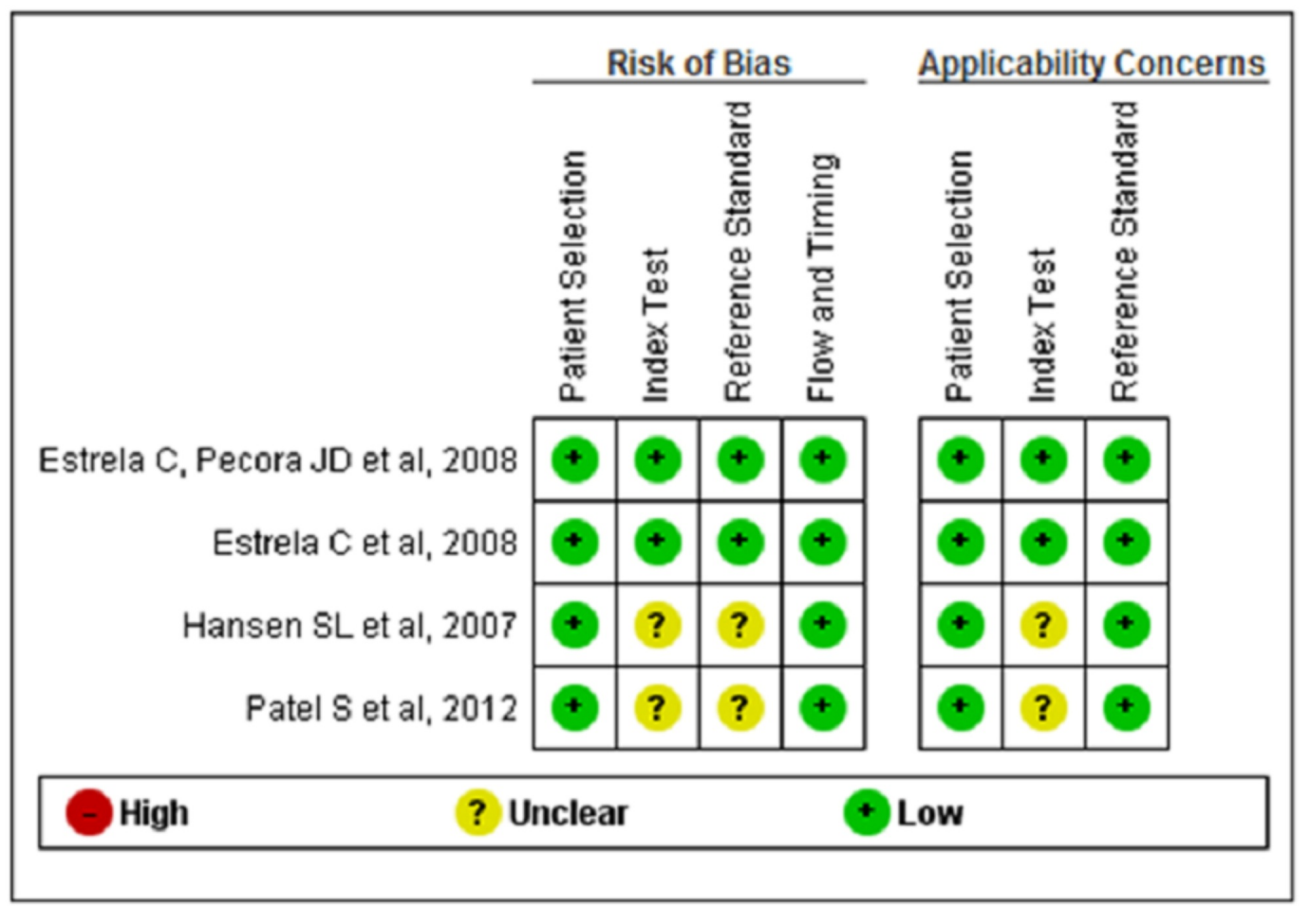
Risk of bias and applicability concerns summary: review authors' judgments about each domain for each of the included studies

**Figure 3:**
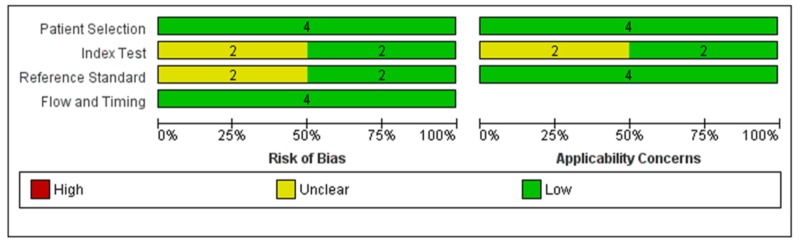
Risk of bias and applicability concerns graph: review authors' judgments about each domain presented as percentages across included studies

We used the recommended approach for assessing the risk of bias using QUADAS. We completed a risk of bias table for each included study and presented graphically by study and by domain across all studies (Figures 2, 3) [13-16]. Two studies had a moderate risk of bias, whereas two studies had a low risk of bias.

Results of the search

The characteristic of the studies included the following eight entries: study ID, clinical features and settings, participants, study design, target condition, index and comparator test, manufacturer and technical details, and follow-up. This information is presented for each study in Table 1. Similarly, the number of studies excluded with the reason for exclusion is presented in Table 2.

**Table 1:** Characteristics of Included studies CBCT: cone-beam computed tomography

Characteristics	Details			
Study ID	Lofthag-Hansen et al. [13]	Estrela et al. [14]	Estrela et al. [15]	Patel et al. [16]
Clinical features and settings	Patients had at least one tooth with a history of secondary and primary endodontic infections, which is tender on percussion. Pre-diagnostic radiographs (periapical and CBCT). Root canal treatment was done in a university clinical set-up	Performed	Performed	Performed
Participants	46 teeth of 36 patients; mean age: 50 years; men: 36.2%, women: 63.8%	1,508 teeth of 888 patients; mean age: 0 years; men: 41%, women: 59%	1,014 teeth of 596 patients; mean age: 54 years; men: 40.4%, women: 59.6%	123 teeth of 99 patients; mean age: 44.5 years
Study design	A single group with a periapical lesion consecutively enrolled; directly compared pre- and post-periapical radiograph and CBCT; participants identified prospectively and all received both radiographs	A single group with a periapical lesion consecutively enrolled; directly compared pre- and post-periapical radiograph and CBCT; participants identified retrospectively, and all received both radiographs	Performed	A single group with a periapical lesion consecutively enrolled; directly compared pre- and post-periapical radiograph and CBCT; participants identified prospectively and all received both radiographs
Target condition	Periapical lesion due to pulpal pathology	Apical periodontitis	Apical periodontitis	Periapical lesion of inflamed pulpal pathology
Index and comparator test	Periapical radiograph and CBCT. Three specialists in oral and maxillofacial radiology analyzed all radiographs together. First, the intraoral ones were evaluated and, after two weeks, all CBCT images. Later, a direct comparison between intraoral radiographs and CBCT images was performed. Kappa value was not mentioned	Periapical, panoramic radiograph, and CBCT. Three calibrated blinded examiners visualized. The presence of periapical lesion diagnosed by CBCT considered as the standard reference. Kappa value: 0.89-1.00 for periapical, panoramic radiograph, and CBCT	Periapical radiographs and CBCT. Three calibrated blinded observers evaluated all digital images by using the CBCT and periapical. Kappa value: 0.86-0.96 for periapical radiograph and CBCT	Periapical radiographs and CBCT scans. Two calibrated examiners evaluated all the images. Kappa value: 0.7 for periapical radiograph and 0.9 for CBCT
Manufacturer and technical details	Periapical radiographs: parallelling technique and horizontal angle difference of 10 degrees using an Oralix DC (Gendex Corporation, Milwaukee, WI) dental X-ray machine at 65 kV and 7.5 mA. Film distance: 22 cm and exposure time; 0.32-0.5 s with F-speed films (Kodak Insight; Eastman Kodak, Rochester, NY). CBCT operating parameters were 2.0-4.0 mA, 80 kV, exposure time: 17.5 s using sagittal slices (1 mm thick). Images analyzed by Dell Workstation PWS 350 and Dell monitor (size 18 inches) (Dell, Round Rock, TX) with Trinitron tube, 1,024 x 768 pixels (Sony, Tokyo, Japan)	Periapical radiographs: Max S-1 X-ray equipment (J. Morita Corp., Osaka, Japan) with 0.8 mm x 0.8 mm tube focal spot and with Kodak Insight film (Eastman Kodak, Rochester, NY) using a parallel technique. CBCT images: 3D Accuitomo XYZ Slice View Tomograph (model MCT-1; J. Morita Corp.) voxel size of 0.125 x 0.125 x 0.125mm, 12 or 8 bits. Images examined by 3D Tomo x version 1.0.51. Panoramic radiographs: Veraviewepocs panoramic X-ray unit (J. Morita Corp.) with a 0.5 mm x 0.5 mm tube focal spot with Kodak dental films (T-MAT, 15 x 30; Manaus, Brazil)	Performed	Periapical radiographs: dental X-ray machine (Planmeca Prostyle Intra, Helsinki, Finland) using a digital CCD (Schick Technologies, New York, NY) at 66 kV, 7.5 mA, and 0.10 s using parallel. Small-volume (40 mm^3^) CBCT scans (3D Accuitomo F170; J Morita Corp.) at 90 kV, 5.0 mA, and 17.5 s, reformatted (0.125 slice intervals and 1.5 mm slice thickness)
Follow-up	No loss to follow-up or missing or un-interpretable test results	Performed	Performed	Followed up for one year. No loss to follow-up or missing or uninterpretable test results

**Table 2:** Characteristics of excluded studies

Study no.	Author	Reason for exclusion
1	Abella et al. [17]	No endodontic treatment performed
2	Kaya [18]	Compared before and after endodontic treatment with no periapical lesion
3	Gumru [19]	Only a subpopulation was included
4	Rios-Santos et al. [20]	No endodontic treatment performed
5	Raghav et al. [21]	No endodontic treatment performed
6	Levin et al. [22]	Case report
7	Liang et al. [23]	Preoperative radiographs not taken
8	Ridao-Sacie et al. [24]	No endodontic treatment performed
9	Yoshioka et al. [25]	Case report
10	Delano et al. [26]	Animal study
11	Molander et al. [27]	Unable to access
12	Rohlin et al. [28]	Unable to access

Each study evaluated periapical lesions using different methods of evaluation. One study used the PAI based on eight categories qualitatively, which compared 46 teeth of 36 patients and showed that periapical radiograph detected 70% of the periapical lesions and CBCT detected 91.3% of the periapical lesions [13]. Another study evaluated the periapical lesions using the PAI scoring system. It reported that periapical radiograph detected 35.3%, panoramic radiograph detected 17.6%, and CBCT detected 63.3% of the presence of periapical periodontitis [14]. Similarly, another study used a modified PAI scoring system with an additional score of E (i.e., expansion of periapical cortical bone) and D (i.e., destruction of periapical cortical bone); this study showed that periapical radiograph detected 40% and 60% of presence and absence of periapical periodontitis, respectively, compared to CBCT, which detected 61% and 39%, respectively [15]. Another study qualitatively evaluated periapical lesion healing based on six categories, which showed that digital periapical radiograph detected 89.6% and CBCT detected 86.1% of reduced periapical lesions [16]. Summary results, method of evaluation, and outcomes of each study are provided in Table 3. Summary findings across the studies are presented in Table 4.

**Table 3:** Summary of results of included studies CBCT: cone-beam computed tomography

Author/year	Method of evaluation	Results	Outcomes
Lofthag-Hansen et al./2007 [13]	Number of roots, root canals (unfilled and filled), roots involved in a lesion, presence of root canal post, periapical lesion, size of the lesion, the effect on or perforation of the cortical bone plate, the distance between a lesion and mandibular canal/maxillary sinus apex and mandibular canal, expansion of lesion into the maxillary sinus, apical-marginal communication, and marginal bone level	Periapical radiograph detected 69.5% and CBCT detected 91.3% of periapical lesion	The detection of apical periodontitis was considerably higher with CBCT than with periapical radiography
Estrela et al./2008 [14]	Periapical index: normal periapical structures; small changes in the bone structure; changes in the bone structure with some mineral loss; periodontitis with a well-defined radiolucent area; severe periodontitis with exacerbating features	Panoramic detected 17.6% and 82.4%, periapical radiograph detected 35.3% and 64.7%, and CBCT detected 63.3% and 36.7% of presence and absence of apical periodontitis respectively	The prevalence of correct identification apical periodontitis was higher with CBCT in comparison with periapical and panoramic radiographs
Estrela et al./2008 [15]	Intact periapical bone structures; diameter of periapical radiolucency: 0.5-1 mm; diameter of periapical radiolucency: 1-2 mm; diameter of periapical radiolucency: 2-4 mm; diameter of periapical radiolucency: 4-8 mm; diameter of periapical radiolucency 8 mm; score (n) # E (expansion of periapical cortical bone); score (n) # D (destruction of periapical cortical bone)	Periapical radiograph detected 39.5% and 60.5%, and CBCT detected 60.9% and 39.1% of presence and absence of apical periodontitis, respectively	The detection of apical periodontitis was considerably higher with CBCT than with periapical radiography
Patel et al./2012 [16]	Based on six categories of periapical changes: new periapical radiolucency; enlarged periapical radiolucency; unchanged periapical radiolucency; reduced periapical radiolucency; resolved periapical radiolucency; unchanged healthy periapical status	Periapical radiograph identified 89.6% and 10.4%, and CBCT identified 86.1% and 13.9% of reduced and unchanged lesions respectively	Periapical radiolucency revealed a 14-fold higher failure rate when assessed using CBCT (17.6%) compared with periapical radiographs (1.3%)

**Table 4:** Summary of findings across studies - the efficacy of various imaging methods in detecting lesions CBCT: cone-beam computed tomography

Study ID	Periapical radiograph	Panoramic radiograph	CBCT
Lofthag-Hansen et al. [13]	69.5%	-	91.3%
Estrela et al. [14]	35.3%	17.6%	63.3%
Estrela et al. [15]	39.5%	-	60.9%
Patel et al. [16]	10.4%	-	13.9%

Only one study reported the sensitivity and specificity for periapical and panoramic radiographs by keeping CBCT as the standard reference. The sensitivity for periapical and panoramic radiograph was 55% and 28%, respectively; specificity and positive predictive values (PPV) ranged from 0.96 to 1.00, and negative predictive values (NPV) ranged from 0.35 to 0.65. The overall accuracy for periapical and panoramic radiographs was 70% and 54%, respectively [14].

Summary of the main results

One study examined 36 patients who had undergone endodontic treatment. A total of 46 teeth were included in the study and were subjected to periapical radiography and CBCT. Periapical lesions were detected in the same 32 teeth by periapical radiograph and CBCT. An additional 10 teeth were detected using CBCT. With both techniques analyzed together, all observers agreed that the CBCT images in those 32 cases provided clinically relevant additional information such as better visualization of the anatomy of the roots and root canals, improved understanding of the location of lesions, and the relation of the lesions with the surrounding structures. CBCT detected more lesions compared to periapical radiographs [13].

Another study examined 888 consecutive patients for the detection of periapical lesions using CBCT, periapical radiography, and panoramic radiography after endodontic treatment. Radiographs were taken, and the assessment was based on the PAI. CBCT tended to offer greater scores than periapical and panoramic radiographs. The limitations of conventional radiographs include compression of 3D anatomy into a 2D shadowgraph causing geometric distortion and a high possibility for false-negative diagnosis [14].

An additional study gave a new PAI based on CBCT. This study included 596 patients; all patients had one or more endodontically treated teeth. Periapical radiographs and CBCT images were taken to detect the presence of a periapical lesion. All the images were subjected to the CBCT PAI, which has some advantages for clinical application. The goal of this index is to offer a method based on the interpretation of high-resolution images that can provide a more precise measurement of AP extension, minimizing observer interference, and increasing the reliability of research results. CBCT was shown to be more accurate than periapical radiography in the diagnosis of AP [15]. Another study included 99 patients who were assessed one year after endodontic treatment by a single operator that compared periapical radiographs and CBCT. The images were assessed by the PAI using six categories for the detection of periapical lesions. The study revealed lower healed and healing rates for root canal treatment assessed with CBCT than with periapical radiographs [16].

Strengths and weaknesses of the review

The review included retrospective and prospective studies and excluded case reports, case series, animal studies, in-vitro, and ex-vivo studies. Since human in-vivo studies have been reviewed, there is applicability for clinical practice. We have taken steps to minimize bias in every step of the review. We searched databases and trial registries with no language limitations to identify all the relevant reports. We assessed the methodological quality of included studies using QUADAS, a 14-item tool for the quality assessment of studies of diagnostic accuracy that provides detailed information on the risk of bias and concerns regarding applicability. All included studies had good methodological quality. Though each study evaluated periapical lesion using different methods with different criteria, the detection of presence or absence of periapical lesion was reported in almost all studies with the standard reference being CBCT. Only one study reported the sensitivity, specificity, PPV, NPV, and accuracy for the periapical and panoramic radiographs [14]. Therefore, no statistical analysis and data synthesis, sensitivity analysis, and investigation of heterogeneity had been performed. This review failed to search other databases such as Excerpta Medica dataBASE (EMBASE) and EBSCO.

The available evidence is from a range of countries and is applicable to patients older than 16 years. Identified studies did not include patients without periapical lesions and endodontic treatments. The results of this review may or may not be generalizable to these groups. All included studies were conducted in university clinics with a single operator, and radiographs were assessed by experts with minimal disagreement. Thus, the generalizability of this review results is possible. Since all included studies reported the manufacturer and technical details for periapical radiograph, panoramic radiograph, and CBCT, there was no limitation for external validity.

## Conclusions

Based on this review, all studies with good quality of evidence demonstrated that CBCT provided better detection of periapical lesions after endodontic treatment, followed by periapical and panoramic radiography. The success of CBCT depends on the familiarity of the practitioner with the technique and the assessment of the images. Thus, a periapical radiograph would be an alternative for CBCT with better visualization and accuracy. In the future, research should be aimed at the better matching of groups and variables such as operator experience and familiarity to validate the findings of the radiographic imaging.

## Appendices

**Table 5:** Search strategies

Search	Add to builder	Query	Items found	Time
#1	Add	Search endodontics	29,144	12:06:05
#2	Add	Search apical periodontitis	4,826	12:06:23
#3	Add	Search chronic apical periodontitis	772	12:07:08
#4	Add	Search periapical periodontitis	4,355	12:07:26
#5	Add	Search apical lesion	1,044	12:07:50
#6	Add	Search periapical disease	7,088	12:08:12
#7	Add	Search root canal therapy	18,668	12:08:25
#8	Add	Search root filled teeth	2,078	12:09:00
#9	Add	Search in vivo	6,54,476	12:09:10
#10	Add	Search periapical radiography	2,617	12:09:32
#11	Add	Search radiography	9,10,866	12:09:41
#12	Add	Search dental digital radiography	2,506	12:10:08
#13	Add	Search dental panoramic radiography	3,889	12:10:24
#14	Add	Search cone-beam computed tomography endodontics	292	12:10:38
#15	Add	Search periapical healing	1,089	12:10:59
#16	Add	Search apical healing	1,169	12:11:05
#17	Add	Search periapical status	412	12:11:20
#18	Add	Search periapical index scoring system	10	12:11:37
#19	Add	Search orstavik periapical index	18	12:11:48
#20	Add	Search treatment outcome	8,18,237	12:12:02
#21	Add	Search endodontic treatment outcome	980	12:12:11
#22	Add	Search ((((((((endodontics) OR apical periodontitis) OR chronic apical periodontitis) OR periapical periodontitis) OR apical lesion) OR periapical disease) OR root canal therapy) OR root filled teeth) OR in vivo	6,89,663	12:12:45
#23	Add	Search ((dental digital radiography) OR dental panoramic radiography) OR cone beam computed tomography endodontics	6,230	12:13:07
#24	Add	Search (((((periapical healing) OR apical healing) OR periapical status) OR periapical index scoring system) OR orstavik periapical index) OR endodontic treatment outcome	2,990	12:13:35
#25	Add	Search ((((((((((((endodontics) OR apical periodontitis) OR chronic apical periodontitis) OR periapical periodontitis) OR apical lesion) OR periapical disease) OR root canal therapy) OR root filled teeth) OR in vivo)) AND (((dental digital radiography) OR dental panoramic radiography) OR cone beam computed tomography endodontics)) AND ((((((periapical healing) OR apical healing) OR periapical status) OR periapical index scoring system) OR orstavik periapical index) OR endodontic treatment outcome)) AND periapical radiography	104	12:14:04
